# Supplementary figures and images for: The quorum sensing regulator RhlR positively controls the expression of the type III secretion system in Pseudomonas aeruginosa PAO1
Source: PLoS One. 2024 Aug 15;19(8):e0307174. doi: 10.1371/journal.pone.0307174 (PMC11326643; doi:10.1371/journal.pone.0307174)

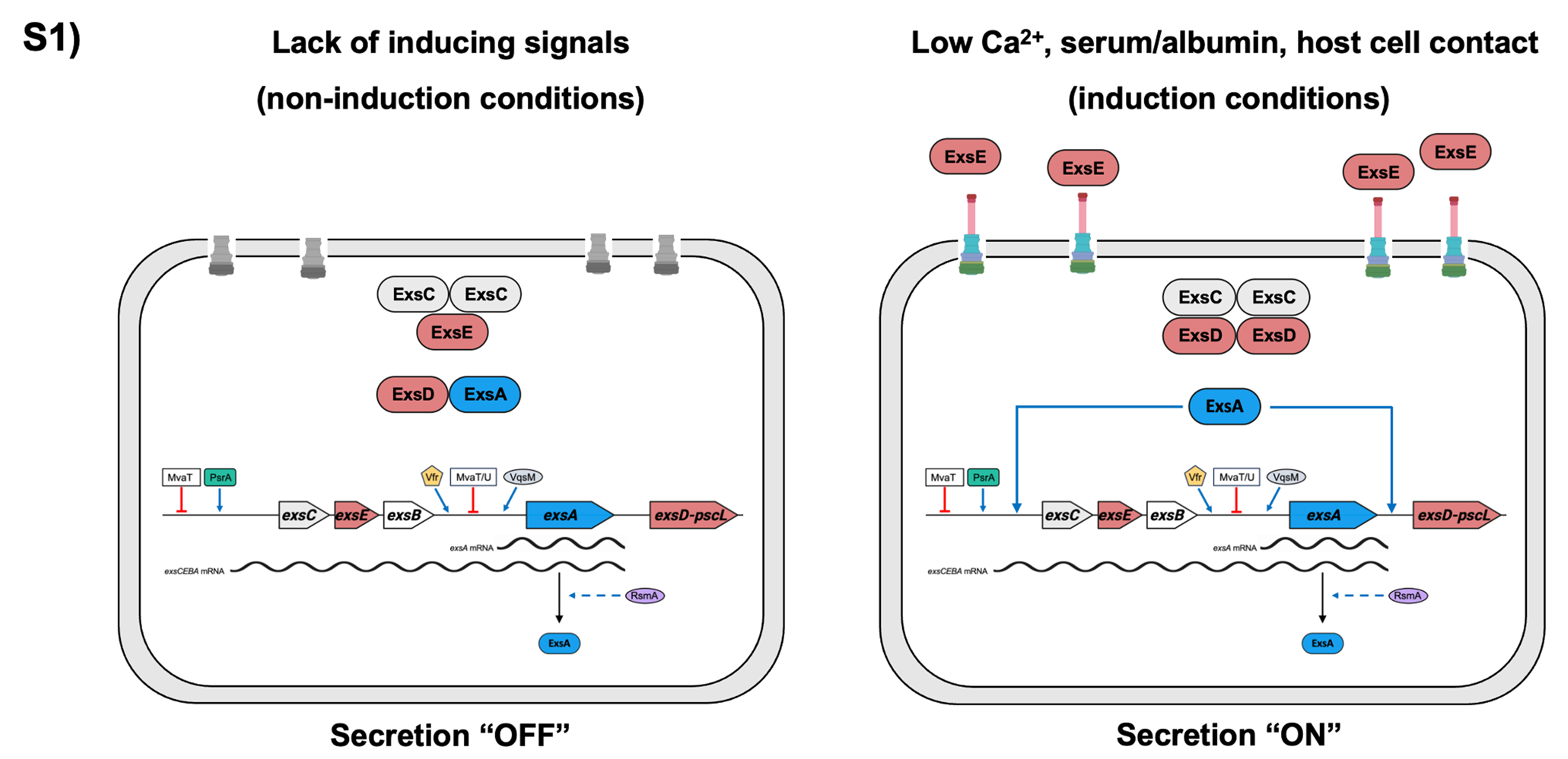

Supplement: S1 Fig — ExsA is the main activator of the T3SS genes. Its activity is controlled by a partner-switching mechanism. During non-induction conditions, ExsD binds to ExsA preventing T3SS activation. Inducing conditions lead to ExsE secretion allowing ExsC to bind ExsD and releasing ExsA, which in turn activates the T3SS expression. Furthermore, exsA expression is controlled by additional transcriptional regulators including PsrA, Vfr, MvaT, VqsM, and the post-transcriptional RsmA regulator. (TIFF) [file pone.0307174.s001.tiff]

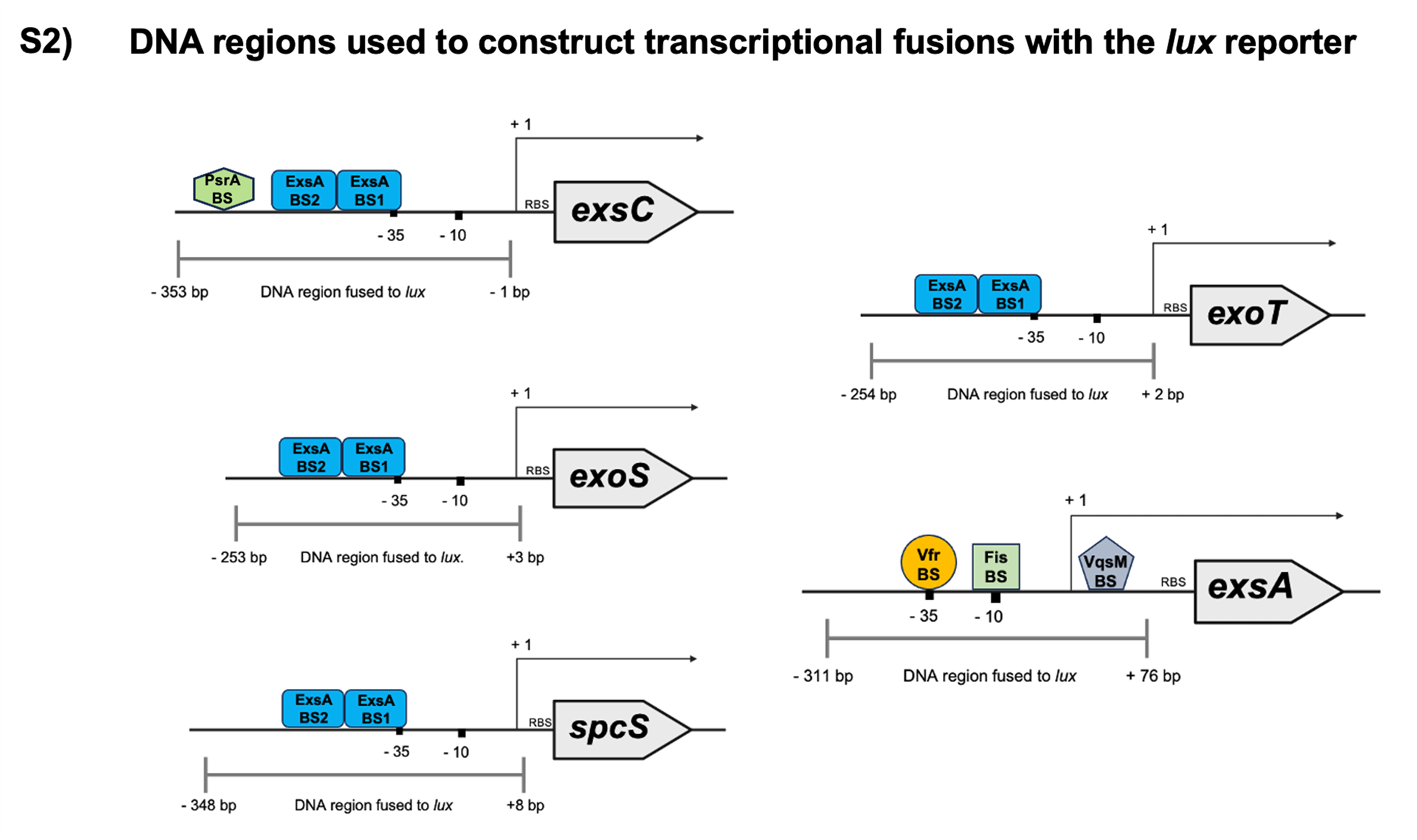

Supplement: S2 Fig — DNA regions include -35 and -10 sequences, ExsA binding sites (BS) and/or sites for additional transcriptional regulators previously reported. Nucleotides in base pair (bp) are indicated according to the transcriptional start site (+1). (TIFF) [file pone.0307174.s002.tiff]

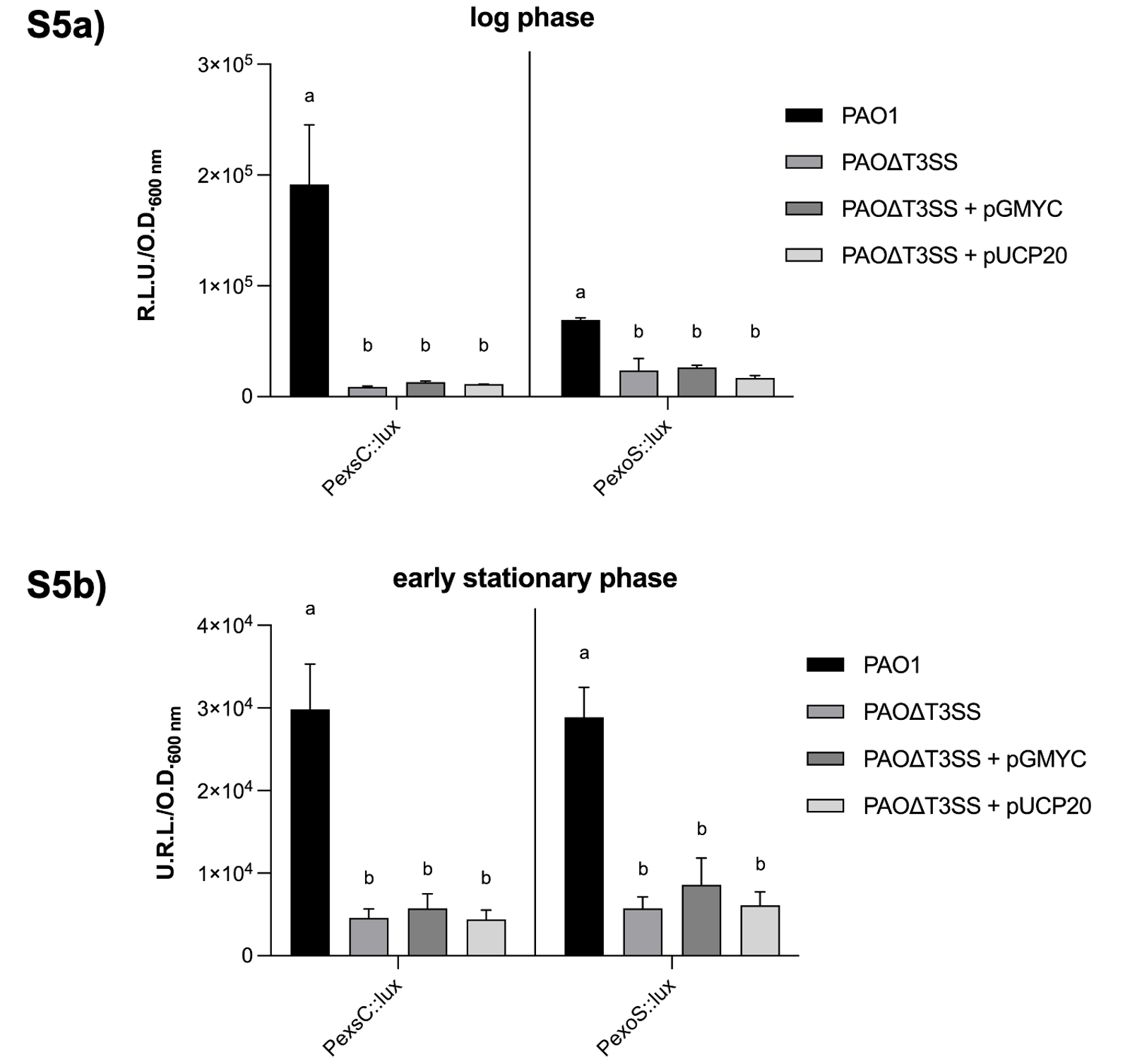

Supplement: S5 Fig — The transcriptional activity of exsCEBA operon (PexsC::lux) and exoS (PexoS::lux) was evaluated in the PAOΔT3SS strain and its derivates with pGMYC or pUCP20 plasmid and in the wild-type PAO1 strain. Strains were incubated in 15 ml of induction medium at 37°C and 225 rpm until reaching an O.D.600 of 0.8 (a) and 2.0 (b). Relative luminescence units (R. L.U.) were quantified and normalized to the O.D.600 at the time of cell collection. Results represent the mean ± S.D. of three biological experiments performed in three replicates each time. Significant differences were obtained by two-way ANOVA and Tukey’s multiple comparison analysis (α = 0.05%). Different letters indicate significant differences, while equal letters indicate no significant differences. (TIFF) [file pone.0307174.s005.tiff]

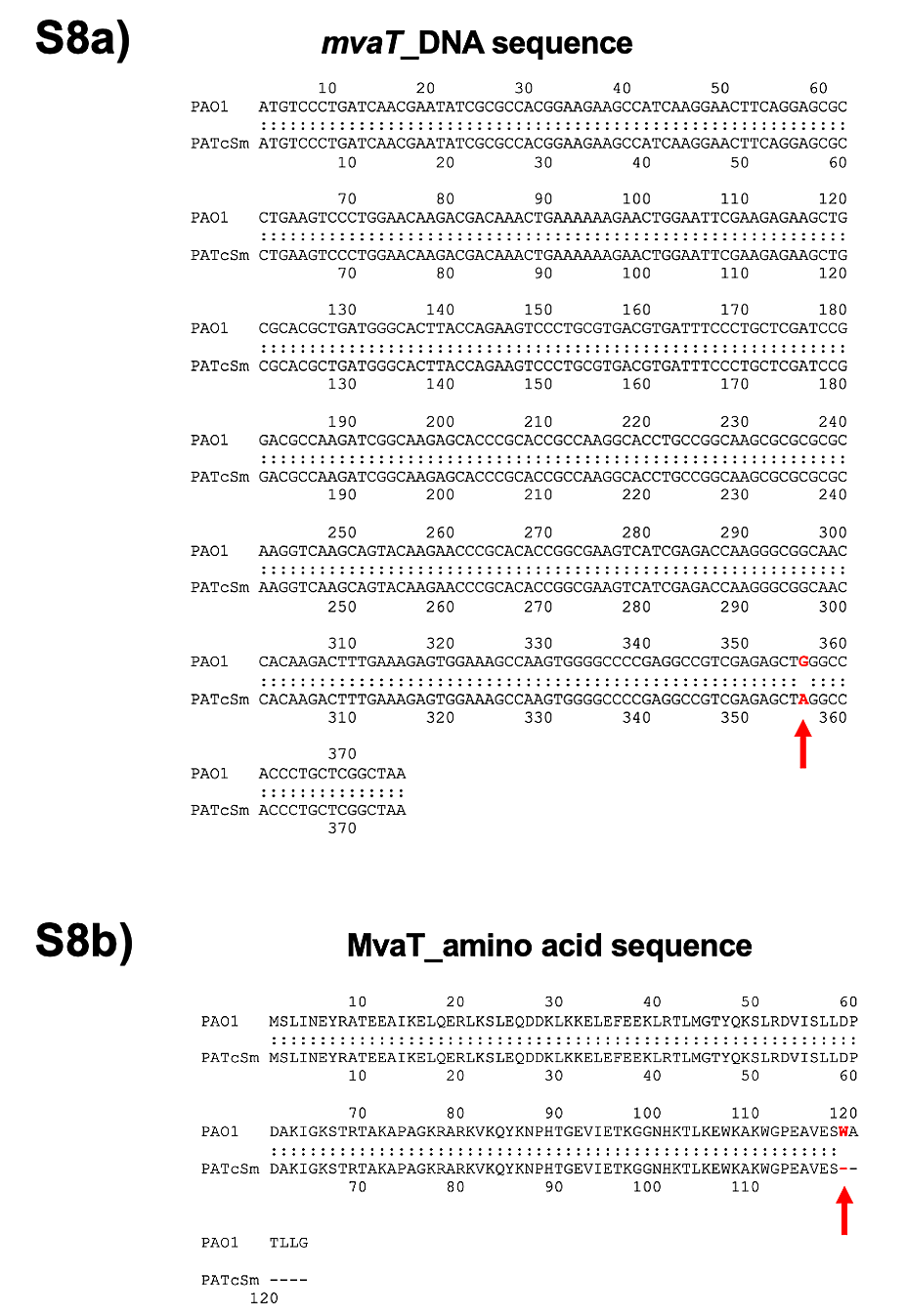

Supplement: S8 Fig — Comparison of the alignment of nucleotide (a) and amino acid (b) sequences of strains PAO1 vs PAOΔlasRTcΔrhlRSm showing a point mutation G355A that generates a stop codon at position Trp119. (TIFF) [file pone.0307174.s008.tiff]
