# Supplementary material for: The quorum sensing regulator RhlR positively controls the expression of the type III secretion system in Pseudomonas aeruginosa PAO1
Source: PLoS One. 2024 Aug 15;19(8):e0307174. doi: 10.1371/journal.pone.0307174 (PMC11326643; doi:10.1371/journal.pone.0307174)
Supplement: S1 Table — (DOCX) [file pone.0307174.s009.docx]

| **S1 Table - Strains and plasmids** | | |
| --- | --- | --- |
| **Strain** | **Characteristics** | **Reference** |
| ***E. coli* DH5α** | φ80Δ*lacZ*ΔM15Δ[*lacZYA*-*argF*] U169 *endA* *recA1 hsdR17 deoR thi-1 supE44* | Invitrogen |
| ***P. aeruginosa*** |  | |
| PAO1 | Wild type strain of the MPAO1 sub-line isolated in Melbourne, Australia from an infected wound. | [1] |
| PAOΔ*rhlR* | MPAO1 derived *rhlR::*Ω mutant, Sm^R^. | [2] |
| PAOΔ*rhlI* | MPAO1 derived *rhlI::*Ω-Flp unmarked mutant. | This work |
| PAOΔ*lasR* | MPAO1 derived *lasR::*Ω mutant, Apc_R_. | This work |
| PAOΔ*lasR*Δ*rhlR* | MPAO1 derived *lasR::*Ω, Apc^R^ and *rhlR::*Ω, Sm^R^ double mutant. | This work |
| PAOΔ*pqsE* | MPAO1 derived *pqsE::*Ω, Gm^R^ mutant. | [3] |
| PAOΔ*rhlI*Δ*pqsE* | MPAO1 derived *rhlI::*Ω-Flp and *pqsE::*Ω, Gm^R^ double mutant. | This work |
| ATCC9027 | Isolated from outer-ear infection. It has a 25.7 kpb deletion that inactive the T3SS. | ATCC |
| PAOΔ*rhlR^Gm^* | MPAO1 derived *rhlR::*Ω mutant, Gm^R^. | [4] |
| PAOΔT3SS | MPAO1 derived *T3SS::*Ω-Flp unmarked mutant. | This work |
| **Plasmids** |  | |
| mini-CTX-lux | Tc^R^; HKBS1 cloning vector. Enables unicopy integrative transcriptional fusions in *Pseudomonas* sp. coupled to the *luxCDABE* luminescence reporter gene cluster. | [5] |
| pCTX | Tc^R^; ~400 bp non-coding DNA fragment cloned into the SmaI site of mini-CTX-lux plasmid, used as luminescence negative control. | This work |
| PexsA::lux | Tc^R^; 388 bp DNA fragment containing the promoter region of the *exsA* gene, from *P. aeruginosa* strain PAO1, cloned into the XhoI and HindIII sites of the mini-CTX-lux. | This work |
| PexoS::lux | Tc^R^; 257 bp DNA fragment containing the promoter region of the *exoS* gene, from *P. aeruginosa* strain PAO1, cloned into the XhoI and HindIII sites of the mini-CTX-lux. | This work |
| PexoT::lux | Tc^R^; 257 bp DNA fragment containing the promoter region of the *exoT* gene, from *P. aeruginosa* strain PAO1, cloned into the XhoI and HindIII sites of the mini-CTX-lux. | This work |
| PexsC::lux | Tc^R^; 353 bp DNA fragment containing the promoter region of the *exsCEBA* operon, from *P. aeruginosa* strain PAO1, cloned into the XhoI and HindIII sites of the mini-CTX-lux. | This work |
| PspcS::lux | Tc^R^; 357 bp DNA fragment containing the promoter region of the *spcS* gene, from *P. aeruginosa* strain PAO1, cloned into the XhoI and HindIII sites of the mini-CTX-lux. | This work |
| pUCP20 | Cb^R^; High copy number expression vector with *plac* promoter able to replicate in *P. aeruginosa* and *E. coli*. | [6] |
| pExsA | Cb^R^; *plac-exsA*. Contains *exsA* gene from PAO1 cloned into pUCP20. | This work |
| pUC2592 | Cb^R^; *plac-PA2592*. Contains *PA2592* gene from PAO1 cloned into pUCP20. | This work |
| pGMYC | Cb^R^; *plac-rhlR*. Contains *rhlR* gene from PAO1 cloned into pUCP20 | [7] |
| pIJ773 | aac(3)IV(Apra^R^) + oriT plasmid | [8] |
| pEX18Gm | Gm^R^; oriT^+^ sacB^+^, gene replacement vector with MCS from pUC18. | [9] |
| pEX-lasR::Apc | Apc^R^; pEX18Gm deletion allele to replace *lasR* from PAO1 with the apramycin resistance marker. | This work |
| pEX-rhlI::Aa | Apc^R^; pEX18Gm deletion allele to replace *rhlI* from PAO1 with the apramycin resistance marker. | [2] |
| pEX-pqsE::Gm | Gm^R^; pEX18Sm deletion allele to replace *pqsE* from PAO1 with the gentamicin resistance marker. | [3] |
| pJET1.2/blunt | Cb^R^, cloning vector unable to replicate in *Pseudomonas aeruginosa*. | Thermo-Fischer |
| pJET1.2_T3SS | Apc^R^; derived from pJET1.2/blunt to replace *exsA*, *exsD*, *pscB* and part of *pscC* from PAO1 with the apramycin resistance marker. | This work |
| pFLP2 | Apc^R^*;* 2.6-kb BamHI–SphI fragment from pALB2 ligated  between the same sites of pPS908. | [9] |
| *Resistance abbreviations: Apc^R^ (Apramycin),Cb^R^ (Carbenicillin) Gm^R^ (Gentamicin), Sm^R^ (Streptomycin), Tc^R^ (Tetracycline). | | |

**References**

1. Holloway BW. Genetic Recombination in *Pseudomonas aeruginosa*. Microbiology. 1955;13: 572–581. doi:10.1099/00221287-13-3-572

2. Morales E, González-Valdez A, Servín-González L, Soberón-Chávez G. *Pseudomonas aeruginosa* quorum-sensing response in the absence of functional LasR and LasI proteins: the case of strain 148, a virulent dolphin isolate. FEMS Microbiology Letters. 2017;364. doi:10.1093/femsle/fnx119

3. García‐Reyes S, Cocotl‐Yañez M, Soto‐Aceves MP, González‐Valdez A, Servín‐González L, Soberón‐Chávez G. PqsR‐independent quorum‐sensing response of *Pseudomonas aeruginosa* ATCC 9027 outlier‐strain reveals new insights on the PqsE effect on RhlR activity. Mol Microbiol. 2021;116: 1113–1123. doi:10.1111/mmi.14797

4. Rahim R, Ochsner UA, Olvera C, Graninger M, Messner P, Lam JS, et al. Cloning and functional characterization of the *Pseudomonas aeruginosa rhlC* gene that encodes rhamnosyltransferase 2, an enzyme responsible for di-rhamnolipid biosynthesis: *rhlC* encodes di-rhamnolipid transferase. Molecular Microbiology. 2001;40: 708–718. doi:10.1046/j.1365-2958.2001.02420.x

5. Becher A, Schweizer H. Integration-proficient *Pseudomonas aeruginosa* vectors for isolation of single-copy chromosomal lacZ and lux gene fusions. Biotechniques. 2000;29: 948–952.

6. Cocotl-Yañez M, Soto-Aceves MP, González-Valdez A, Servín-González L, Soberón-Chávez G. Virulence factors regulation by the quorum-sensing and Rsm systems in the marine strain *Pseudomonas aeruginosa* ID4365, a natural mutant in *lasR*. FEMS Microbiology Letters. 2020;367: fnaa092. doi:10.1093/femsle/fnaa092

7. Medina G, Juárez K, Díaz R, Soberón-Chávez G. Transcriptional regulation of *Pseudomonas aeruginosa* rhlR, encoding a quorum-sensing regulatory protein. Microbiology. 2003;149: 3073–3081. doi:10.1099/mic.0.26282-0

8. Gust B, Challis GL, Fowler K, Kieser T, Chater KF. PCR-targeted *Streptomyces* gene replacement identifies a protein domain needed for biosynthesis of the sesquiterpene soil odor geosmin. Proc Natl Acad Sci USA. 2003;100: 1541–1546. doi:10.1073/pnas.0337542100

9. Hoang TT, Karkhoff-Schweizer RR, Kutchma AJ, Schweizer HP. A broad-host-range Flp-FRT recombination system for site-specific excision of chromosomally-located DNA sequences: application for isolation of unmarked *Pseudomonas aeruginosa* mutants. Gene. 1998;212: 77–86. doi:10.1016/S0378-1119(98)00130-9
