# Supplementary material for: The quorum sensing regulator RhlR positively controls the expression of the type III secretion system in Pseudomonas aeruginosa PAO1
Source: PLoS One. 2024 Aug 15;19(8):e0307174. doi: 10.1371/journal.pone.0307174 (PMC11326643; doi:10.1371/journal.pone.0307174)
Supplement: S2 Table — (DOCX) [file pone.0307174.s010.docx]

| **S2 Table - Oligonucleotides used in this study** | |
| --- | --- |
| **Oligonucleotide name** | **Sequence 5´**🡪**3’** |
| 7460-2016_luxFw  11508-2015_luxRv | CACTATAGGGCGAATTGGGTA  CCTGGCCGTTAATAATGAATG |
| rt-rhlR-F3  rt-rhlR-R2 | CGACCAGCAGAACATCTCC  CCCGTAGTTCTGCATCTGGT |
| 4359-2018_PexsAFw  4360-2018_PexsARv | GAACTCGAGGAGTGCCTGGCAACAGGT  GCGAAGCTTGTCGTACCTTGAATGCCCC |
| 4355-2018_PexoSFw  4356-2018_PexoSRv | GAACTCGAGGCTGAGTACGCTCTCCTCGT  GCGAAGCTTGATGTTTCTCCGCCAGTCTA |
| 4357-2018_PexoTFw  4358-2018_PexoTRv | GAACTCGAGGCACCATAGCAGCAGACGAC  GCGAAGCTTATGTTTCCCCGCCAGTCTAG |
| 384-2019_PexsCEBAFw  387-2019_PexsCEBARv | CCGCTCGAGTGGCCAACACGGTGATCCAGT  CCCAAGCTTCTCAGCGCATGCTAGCACCG |
| 386-2019_PspcSFw  387-2019_PspcSRv | CCGCTCGAGGTGCCGCACCCAAGCGAGCG  CCCAAGCTTAGTCACTGGAGGCAGCCATT |
| 4426-2019_ExsAFw\|  4427-2019_ExsARv | TAAGGATCCAAAACGGAGCGTATTCAT  AAAAAGCTTAATTTGGGCCGATTCTACT |
| 5UpEcIPA2592Fw  3DwBmIPA2592Rv | ACTGAATTCTCCAACTTGTTTCGTTTC  TATGGATCCCCCGGTGAAAGACGCGAA |
| 9522-2014_F-Apra  9523-2014_R-Apra | TATTCCGGGGATCCGTCGAC  TGTAGGCTGGAGCTGCTTC |
| 6709-2015_H3lasRUp  8653-2015_lasR5Apra  6514-2018_lasR3Apra  6710-2015_H3lasRDown | tacaaaaaagcaggctCCGAACTGGAAAAGTGGC  gtcgacggatccccggaataCTTAAACTATTAACCAATC  gaagcagctccagcctacaAATATTCGGCGGAAGTTCG  tacaagaaagctgggtCGAAAACCTGGGCTTCAG |
| 6767_2023_T3SSUp  6768_2023_T3SS3aa  6769_2023T3SS5aa  6770-2023_T3SSDown | tacaaaaaagcaggctGGTGCTGGATGCTGTTGCC  gaagcagctccagcctacaGCAGAGCGAGTCGGCTTCC  gtcgacggatccccggaataTGCCGAGAACCTCGCCCA  tacaagaaagctgggtACTCGTCGATTTCAAGCAGGC |
