# Supplementary material for: The quorum sensing regulator RhlR positively controls the expression of the type III secretion system in Pseudomonas aeruginosa PAO1
Source: PLoS One. 2024 Aug 15;19(8):e0307174. doi: 10.1371/journal.pone.0307174 (PMC11326643; doi:10.1371/journal.pone.0307174)
Supplement: S1 Dataset — (PDF) [file pone.0307174.s011.pdf]

**Fig. 1a**	**R1**	**R2**	**R3**	**R4**	**R5**
ATCC9027	0.0	0.0	0.0	0.0	0.0
PAO1	100.0	100.0	100.0	100.0	100.0
PAOΔ*lasR*	118.4	95.9	74.1	72.8	78.1
PAOΔ*rhlR*	1.4	5.8	2.2	9.3	9.8
PAOΔ*rhlR* + pGMYC	75.0	63.2	91.0	89.5	80.5
PAOΔ*rhlR* + pUCP20	5.8	10.7	7.5	6.8	23.7
PAOΔ*lasR*Δ*rhlR*	0.1	4.0	7.1	2.8	16.0

**Fig. 1b**	**R1**	**R2**	**R3**
ATCC9027	0.0	0.0	0.0
PAO1	100.0	100.0	100.0
PAOΔ*lasR*	131.1	82.2	87.7
PAOΔ*rhlR*	24.1	12.3	4.1
PAOΔ*rhlR* + pGMYC	72.1	103.6	92.0
PAOΔ*rhlR* + pUCP20	3.7	11.5	22.0
PAOΔ*lasR*Δ*rhlR*	10.4	-5.4	-1.1

Fig. 2a	R1	R2	R3
ATCC9027	0.0	0.0	0.0
PAO1	100.0	100.0	100.0
PAOΔ*rhlR*	1.4	5.8	2.2
PAOΔ*rhlR* + pExsA	130.8	121.7	95.4
PAOΔ*rhlR* + pUCP20	5.8	10.7	7.5
PAOΔ*rhlR*_Gm	-1.1	9.2	44.0

Fig. 2b	R1	R2	R3
ATCC9027	0.0	0.0	0.0
PAO1	100.0	100.0	100.0
PAOΔ*rhlR*	24.1	12.3	4.1
PAOΔ*rhlR* + pExsA	159.0	126.0	136.4
PAOΔ*rhlR* + pUCP20	3.7	11.5	22.0
PAOΔ*rhlR*_Gm	-5.8	27.6	55.8

**Fig. 3.- PexsC::lux**	**R1**	**R2**	**R3**
PAO1	155453	129629	165873
PAOΔ*rhlR*	13703	15148	14549
PAOΔ*rhlR* + pGMYC	101926	115112	178234
PAOΔ*rhlR* + pUCP20	15953	12009	22050
PAOΔ*rhlR* + pExsA	349963	402978	411617

**Fig. 3.- PexoS::lux**	**R1**	**R2**	**R3**
PAO1	24131	33635	33772
PAOΔ*rhlR*	11414	11554	12085
PAOΔ*rhlR* + pGMYC	26252	24493	26985
PAOΔ*rhlR* + pUCP20	13239	10244	12258
PAOΔ*rhlR* + pExsA	148458	153803	167190

**Fig. 3.- PspcS::lux**	**R1**	**R2**	**R3**
PAO1	22063	25249	30647
PAOΔ*rhlR*	10819	10703	11990
PAOΔ*rhlR* + pGMYC	21477	28789	21946
PAOΔ*rhlR* + pUCP20	11911	12511	14910
PAOΔ*rhlR* + pExsA	431475	350119	414726

Fig. 4.- Cytotoxicity	R1	R2	R3	R4	R5	R6	R7	R8
ATCC9027	2.1	2.1	7.5	6.4	2.1	6.5	6.6	
PAO1	15.4	11.0	29.3	26.5	21.9	21.2		
PAOΔ*rhlR*	9.3	4.5	9.6	7.6	11.9	8.6	12.5	
PAOΔ*rhlR* + pGMYC	12.3	10.8	11.7	20.0	20.0	25.4	41.9	
PAOΔ*rhlR* + pUCP20	4.5	4.4	12.7	11.4	10.2	12.2	0.6	11.0
PAOΔ*rhlR* + pExsA	33.5	35.4	43.0	46.4	46.5	48.9	32.9	33.0

**Fig. 5a**	**R1**	**R2**	**R3**	**R4**	**R5**
ATCC9027	0.0	0.0	0.0	0.0	0.0
PAO1	100.0	100.0	100.0	100.0	100.0
PAOΔ*rhlR*	1.4	5.8	2.2	9.3	9.8
PAOΔ*rhlI*	93.0	135.5	136.4	76.3	67.5
PAOΔ*pqsE*	141.0	110.7	120.3	114.1	105.8
PAOΔ*rhlI*Δ*pqsE*	59.6	93.6	133.8	47.0	52.0

**Fig. 5b**	**R1**	**R2**	**R3**
ATCC9027	0.0	0.0	0.0
PAO1	100.0	100.0	100.0
PAOΔ*rhlR*	24.1	12.3	4.1
PAOΔ*rhlI*	106.3	79.3	69.2
PAOΔ*pqsE*	105.3	167	121.7
PAOΔ*rhlI*Δ*pqsE*	138.6	123.7	60.8

**Fig. 6.- early stationary phase**	**PAO1**			**PAOΔ*rhlI***		
	**R1**	**R2**	**R3**	**R1**	**R2**	**R3**
PexsC::lux	26870	36157	38585	89381	63924	86661
PexoS::lux	25329	25324	24314	39089	44479	30263
PexoT::lux	13922	7024	11847	29433	20722	23999

**Fig. 7a_log phase**	**R1**	**R2**	**R3**	**R4**	**R5**	**R6**
ATCC9027	0.0	0.0	0.0	0.0	0.0	0.0
PAO1	100.0	100.0	100.0	100.0	100.0	100.0
PAOΔ*rhlR*	5.8	2.2	9.3	9.8	2.2	2.8
PAOΔ*rhlR* + pGMYC	75.0	86.6	91.0	89.5	80.5	87.6
PAOΔ*rhlR* + pUCP20	10.7	7.5	6.8	23.7	5.8	12.8
PAOΔ*rhlR* + pUC2592	17.1	4.9	35.4	59.2	17.2	25.1

**Fig. 7b_early stationary phase**	**R1**	**R2**	**R3**	**R4**
ATCC9027	0.0	0.0	0.0	0.0
PAO1	100.0	100.0	100.0	100.0
PAOΔ*rhlR*	24.1	12.3	4.1	5.0
PAOΔ*rhlR* + pGMYC	72.1	103.6	92.0	64.6
PAOΔ*rhlR* + pUCP20	3.7	11.5	22.0	3.9
PAOΔ*rhlR* + pUC2592	31.2	29.3	51.4	44.8

Fig. 8.- Pyocyanin	R1	R2	R3
ATCC9027	0.005	0.003	0.001
PAO1	0.05	0.043	0.049
PAOΔ*lasR*	0.037	0.035	0.031
PAOΔ*rhlR*	0.007	0.007	0.002
PAOΔ*rhlR* + pGMYC	0.098	0.078	0.079
PAOΔ*rhlR* + pUCP20	0.009	0.008	0.002
PAOΔ*rhlR* + pExsA	0.01	0.007	0.005
PAOΔ*lasR*Δ*rhlR*	0.003	0.008	0.006
PAOΔ*rhlI*	0.01	0.017	0.009
PAOΔ*pqsE*	0.002	0.005	0.002
PAOΔ*rhlI*Δ*pqsE*	0.002	0.005	0.004

Fig. S4_LB non-induced	R1	R2	R3
pCTX	16813.3	15041.1	13998.3
PexsC::lux	82681.9	64125.8	52349.8
PexsA::lux	32011.1	34900.4	27011.1
PexoS::lux	33801.3	30848.2	27880.4
PspcS::lux	22270.1	20890.8	15090.1

Fig. S4_LB induced	R1	R2	R3
pCTX	16786.8	14701.8	15918.6
PexsC::lux	199646.8	186797.3	194704.3
PexsA::lux	29662.5	31126.3	26796.9
PexoS::lux	67483.4	40460.5	70584.0
PspcS::lux	72671.7	52017.8	60597.7

Fig.S5_ log phase	PAO1			PAOΔT3SS			PAOΔT3SS + pGMYC			PAOΔT3SS + pUCP20		
	R1	R2	R3	R1	R2	R3	R1	R2	R3	R1	R2	R3
PexsC::lux	155453	253521	165873	8019	8994	9473	13467	13898	12363	11457	11145	11236
PexoS::lux	68401	68026	71511	15705	19778	35746	25337	28787	24800	16922	19120	14538

Fig.S5_ early stationary phase	PAO1			PAOΔT3SS			PAOΔT3SS + pGMYC			PAOΔT3SS + pUCP20		
	**R1**	**R2**	**R3**	**R1**	**R2**	**R3**	**R1**	**R2**	**R3**	**R1**	**R2**	**R3**
PexsC::lux	26870	36157	26484	5486	3393	4923	7751	4555	4879	5237	3195	4870
PexoS::lux	25329	28714	32593	5675	4375	7155	12324	6717	6674	7585	4380	6413

**Fig. S6**	**R1**	**R2**	**R3**
PAO1	155453	129629	165873
PAOΔ*rhlI*	169457	175219	297822
PAOΔ*pqsE*	193986	287385	250133
PAOΔ*rhlI*Δ*pqsE*	144405	118320	126195
