# Supplementary material for: The quorum sensing regulator RhlR positively controls the expression of the type III secretion system in Pseudomonas aeruginosa PAO1
Source: PLoS One. 2024 Aug 15;19(8):e0307174. doi: 10.1371/journal.pone.0307174 (PMC11326643; doi:10.1371/journal.pone.0307174)

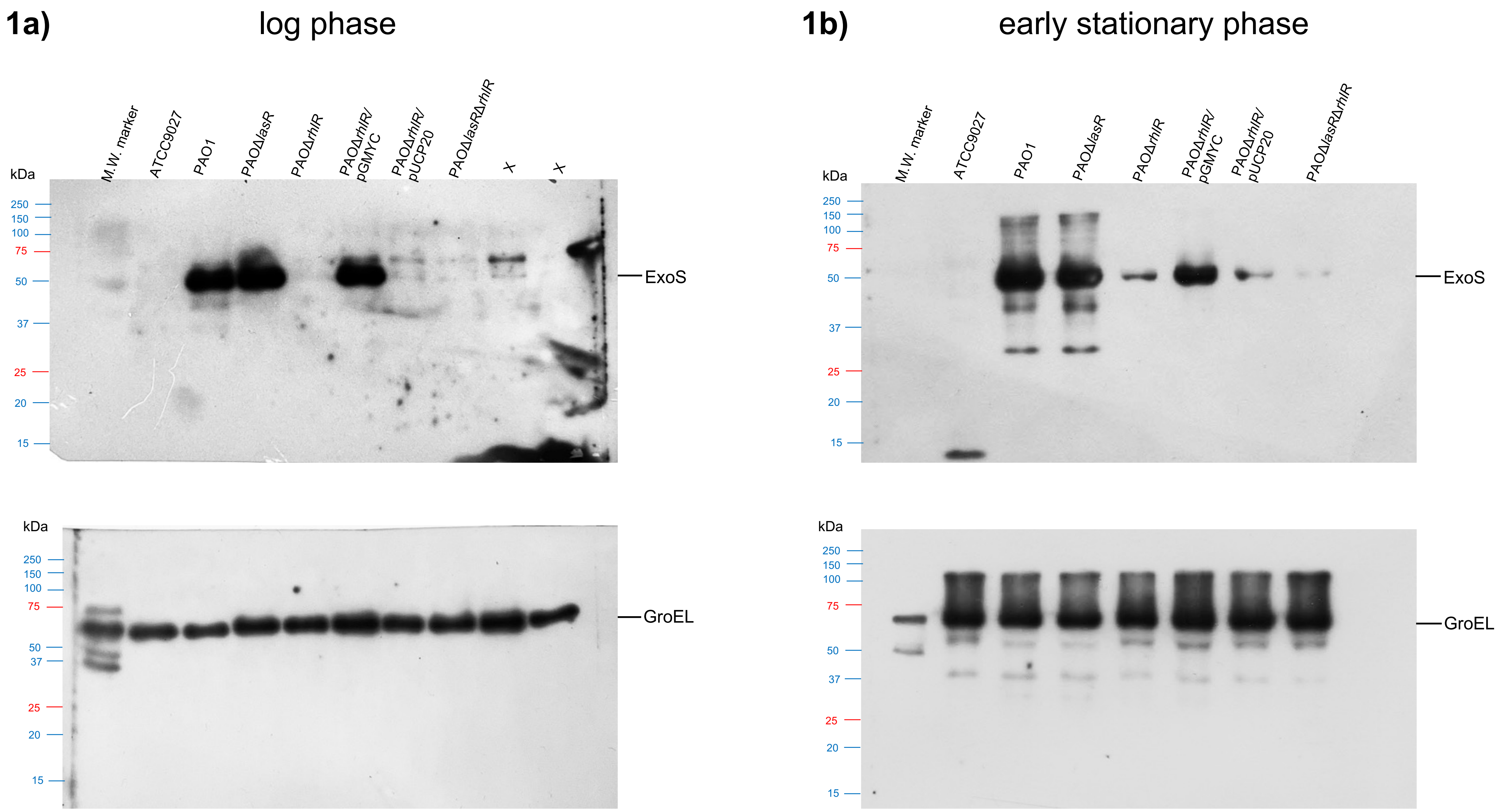
1a)
log phase
kDa
M.W. marker
ATCC9027
PAO1
PAOΔlasR
PAOΔrhlR
PAOΔrhlR/ pGMYC
PAOΔrhlR/ pUCP20
PAOΔlasRΔrhlR
X
X
250
150
100
75
50
37
25
20
15
ExoS
GroEL
1b)
early stationary phase
kDa
M.W. marker
ATCC9027
PAO1
PAOΔlasR
PAOΔrhlR
PAOΔrhlR/ pGMYC
PAOΔrhlR/ pUCP20
PAOΔlasRΔrhlR
250
150
100
75
50
37
25
20
15
ExoS
GroEL

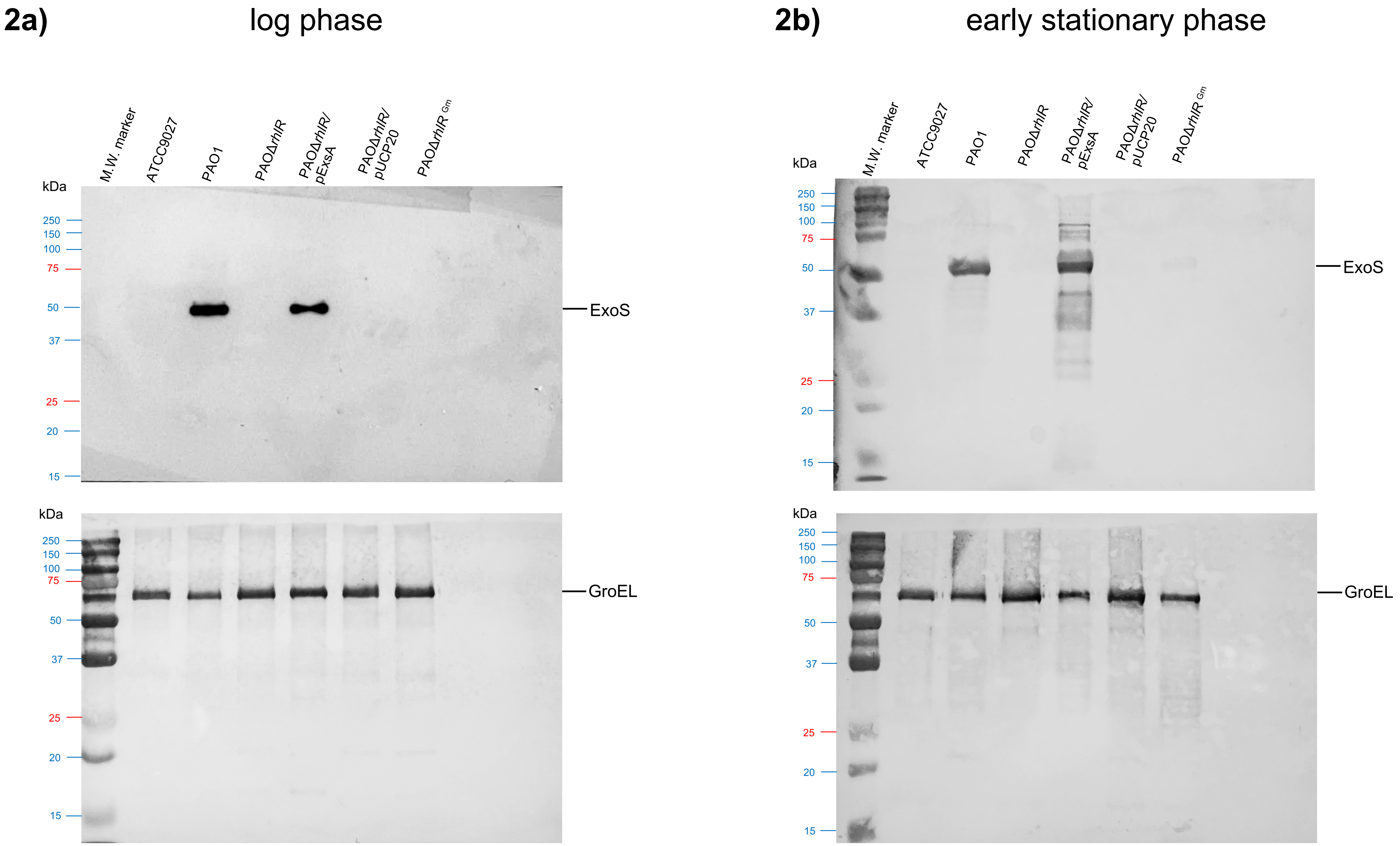

2a)
log phase
M.W. marker
ATCC9027
PAO1
PAOΔrhlR
PAOΔrhlR/ pExsA
PAOΔrhlR/ pUCP20
PAOΔrhlR Gm
kDa
250
150
100
75
50
37
25
20
15
ExoS
GroEL
2b)
early stationary phase

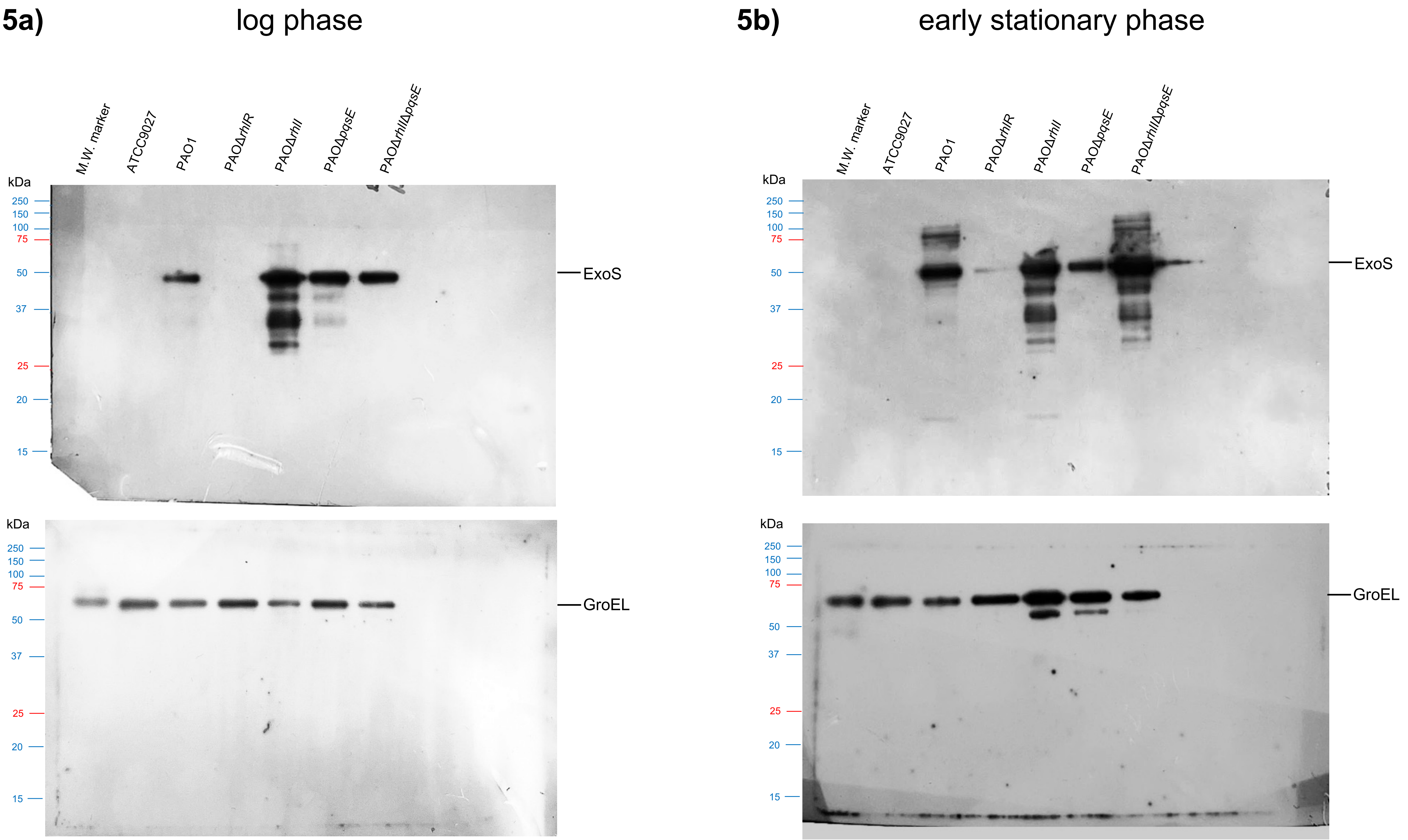
5a)
log phase
M.W. marker
ATCC9027
PAO1
PAOΔrhlR
PAOΔrhlI
PAOΔpqsE
PAOΔrhlIΔpqsE
kDa
250
150
100
75
50
37
25
20
15
ExoS
GroEL
5b)
early stationary phase
M.W. marker
ATCC9027
PAO1
PAOΔrhlR
PAOΔrhlI
PAOΔpqsE
PAOΔrhlIΔpqsE
kDa
250
150
100
75
50
37
25
20
15
ExoS
GroEL

7a) log phase

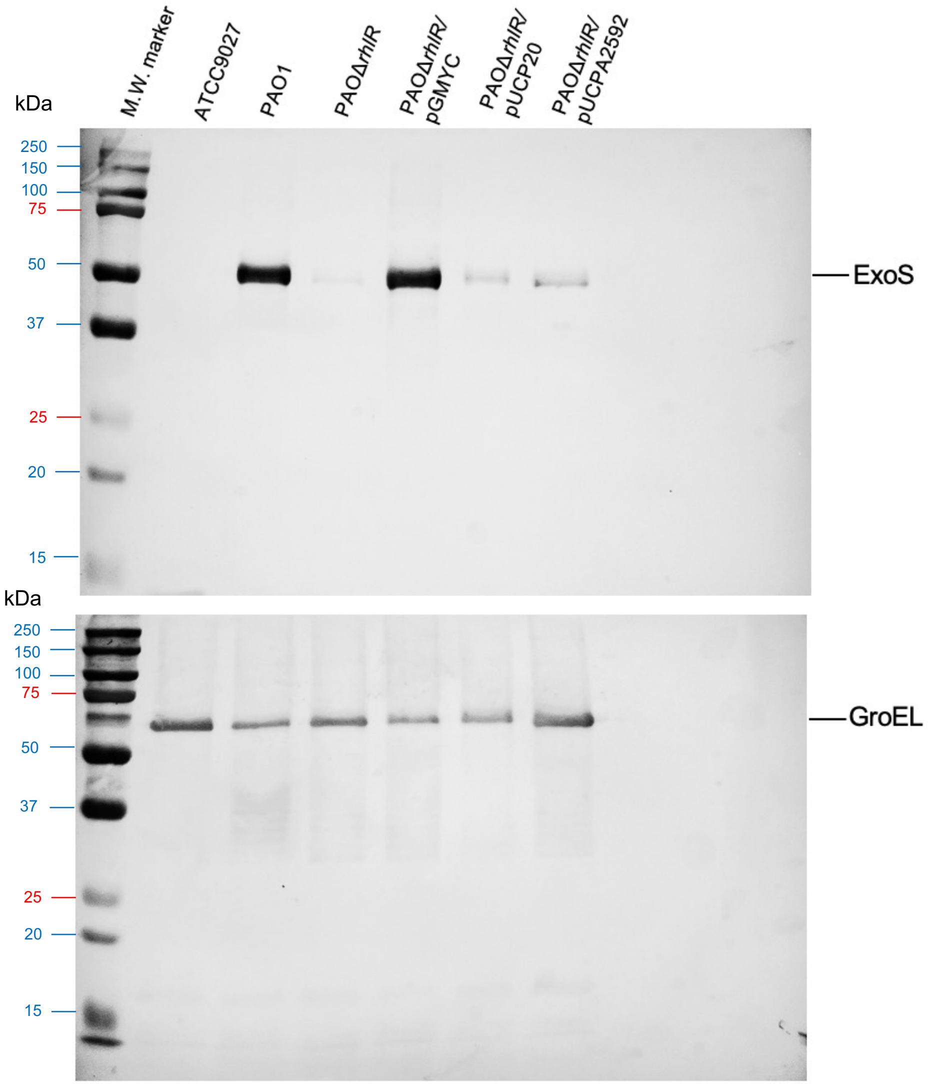

7b) early stationary phase

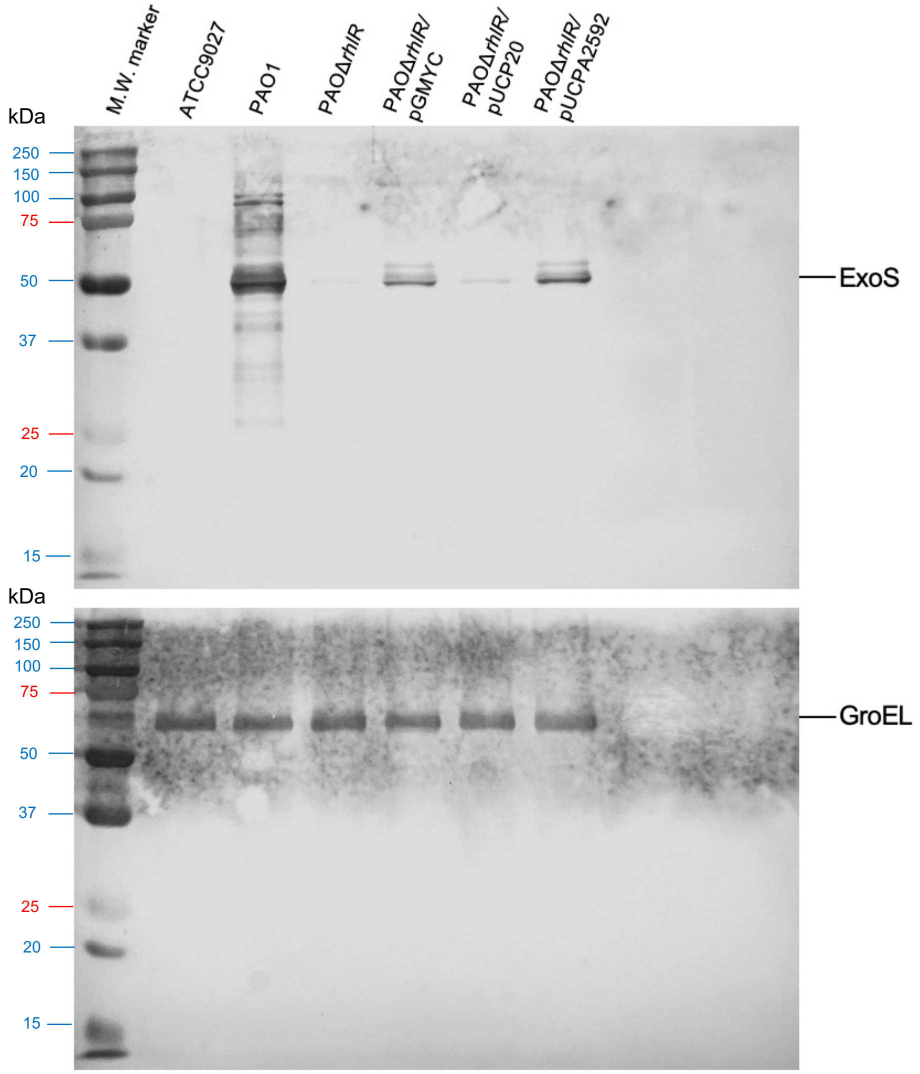

S3)

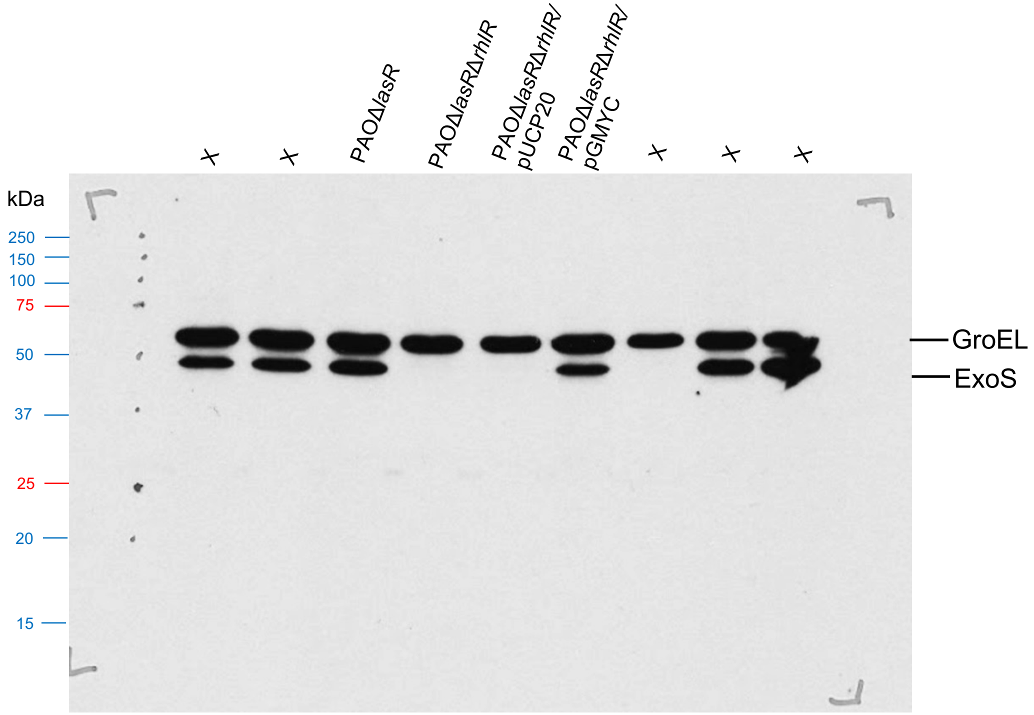
PAOΔlasR
PAOΔlasRΔrhlR
PAOΔlasRΔrhlR/
pUCP20
PAOΔlasRΔrhlR/
pGMYC
kDa
250
150
100
75
50
37
25
20
15
GroEL
ExoS

S7a) LB non-induced

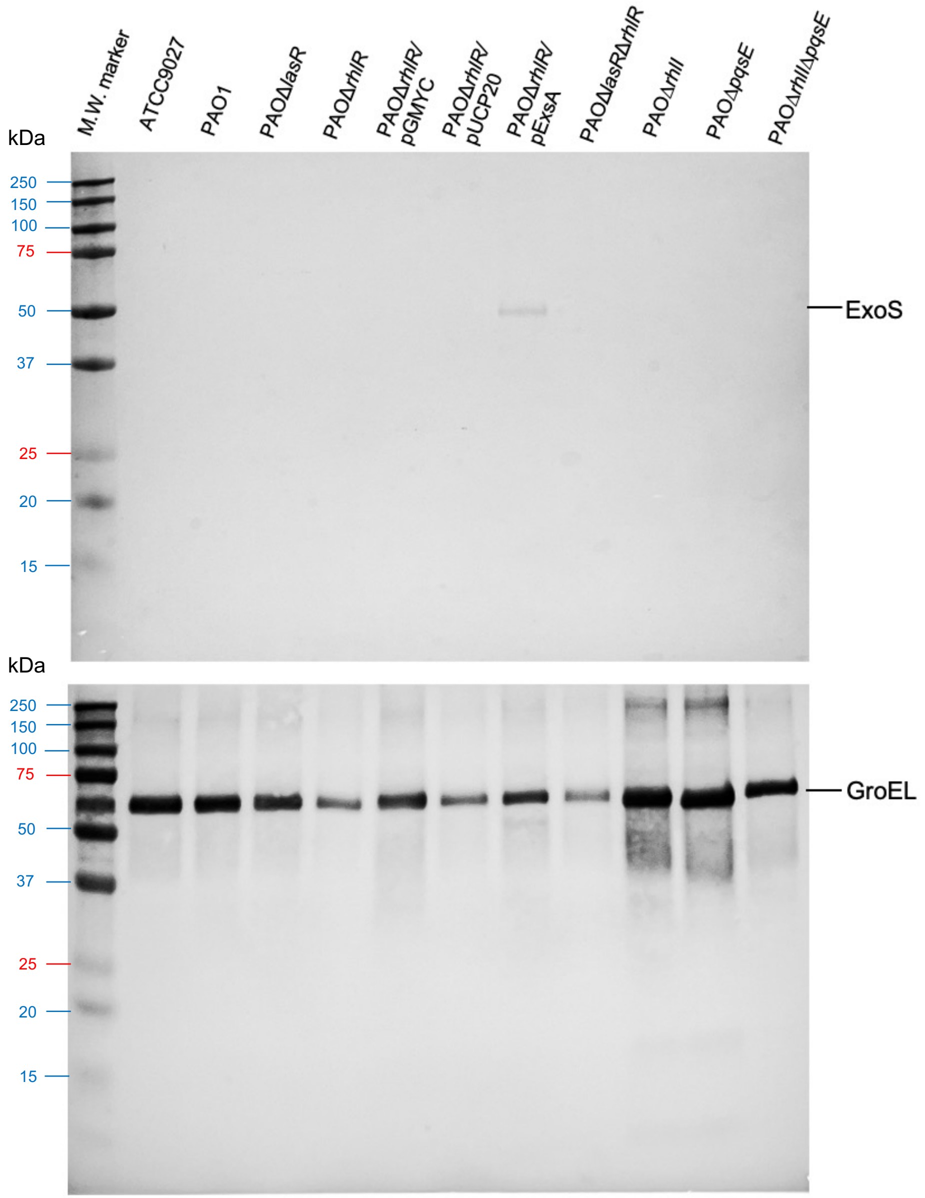

S7a) LB induced

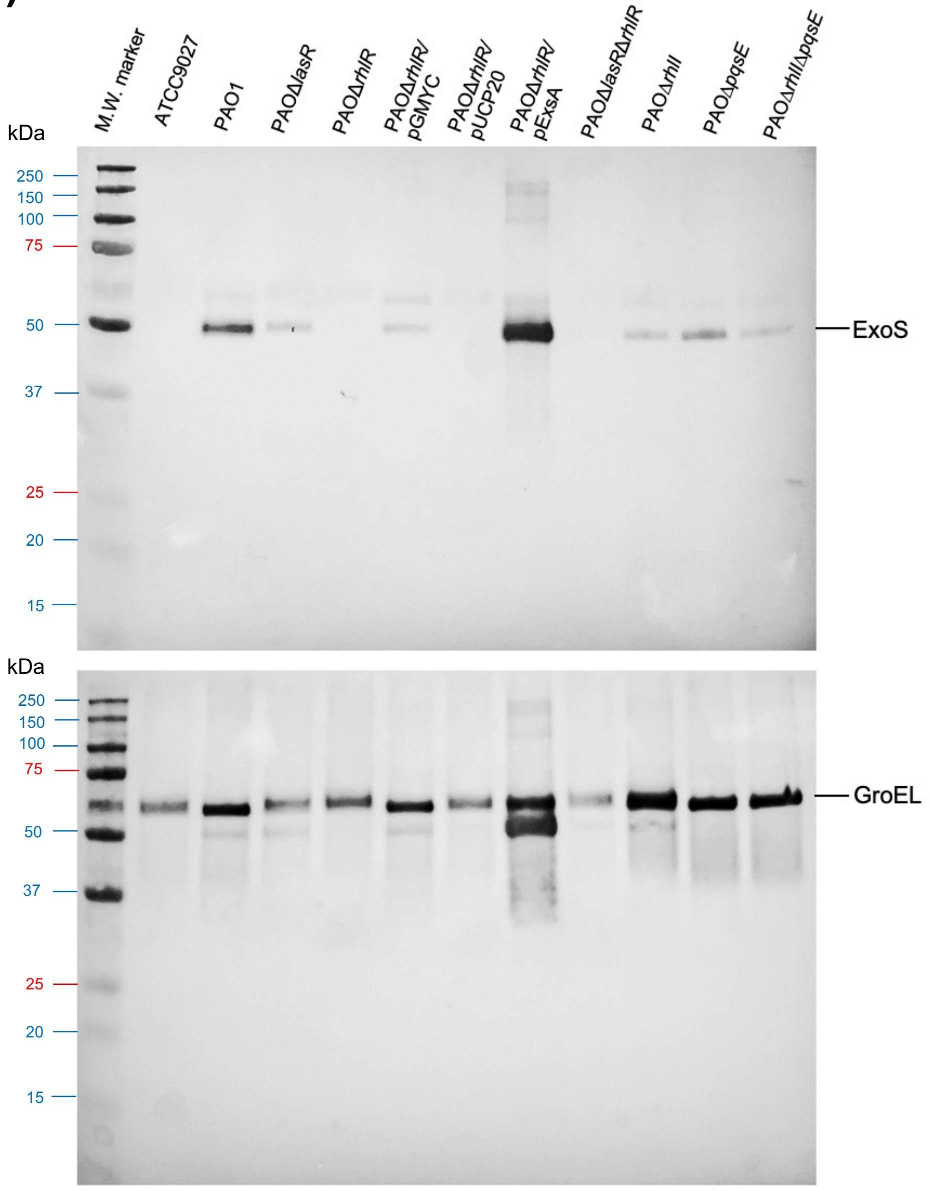

Supplement: S1 Raw images — (PDF) [file pone.0307174.s012.pdf]
