# Supplementary material for: The quorum sensing regulator RhlR positively controls the expression of the type III secretion system in Pseudomonas aeruginosa PAO1
Source: PLoS One. 2024 Aug 15;19(8):e0307174. doi: 10.1371/journal.pone.0307174 (PMC11326643; doi:10.1371/journal.pone.0307174)
Supplement: Attachment — Submitted filename: Response to Reviewers.docx [file pone.0307174.s013.docx]

April 2^nd^, 2024

Rajesh P. Shastry, Ph.D

Academic Editor

PLOS ONE

In this document, we respond to the comments and suggestions made by the reviewers for the manuscript PONE-D-24-00964 entitled ‘The quorum sensing regulator RhlR positively controls the expression of the type III secretion system in *Pseudomonas aeruginosa* PAO1’ by Luis Fernando Montelongo-Martínez, Miguel Díaz-Guerrero, Verónica Roxana Flores-Vega, Martín Paolo Soto-Aceves, Roberto Rosales-Reyes, Bertha González-Pedrajo, Gloria Soberón-Chávez, Miguel Cocotl-Yañez.

Best regards

Miguel Cocotl-Yañez

Corresponding author.

Reviewers' comments:

Reviewer's Responses to Questions

Comments to the Author

1. Is the manuscript technically sound, and do the data support the conclusions?

The manuscript must describe a technically sound piece of scientific research with data that supports the conclusions. Experiments must have been conducted rigorously, with appropriate controls, replication, and sample sizes. The conclusions must be drawn appropriately based on the data presented.

Reviewer #1: Yes

Reviewer #2: Yes

2. Has the statistical analysis been performed appropriately and rigorously?

Reviewer #1: No. We carried out an additional statistical analysis to be more rigorous with our results, this analysis has been included in the revised version.

Reviewer #2: Yes

3. Have the authors made all data underlying the findings in their manuscript fully available?

The PLOS Data policy requires authors to make all data underlying the findings described in their manuscript fully available without restriction, with rare exception (please refer to the Data Availability Statement in the manuscript PDF file). The data should be provided as part of the manuscript or its supporting information, or deposited to a public repository. For example, in addition to summary statistics, the data points behind means, medians and variance measures should be available. If there are restrictions on publicly sharing data—e.g. participant privacy or use of data from a third party—those must be specified.

Reviewer #1: Yes

Reviewer #2: Yes

4. Is the manuscript presented in an intelligible fashion and written in standard English?

PLOS ONE does not copyedit accepted manuscripts, so the language in submitted articles must be clear, correct, and unambiguous. Any typographical or grammatical errors should be corrected at revision, so please note any specific errors here.

Reviewer #1: Yes

Reviewer #2: Yes

5. Review Comments to the Author

Please use the space provided to explain your answers to the questions above. You may also include additional comments for the author, including concerns about dual publication, research ethics, or publication ethics. (Please upload your review as an attachment if it exceeds 20,000 characters)

Reviewer #1: The current study investigates the QS essential proteins namely Las, Rhl, and Pqs QS receptors, and inducers' role in the T3SS activation in P. aureginosa. I find these researches that correlate the QS activation to other bacterial activity is an essential area and unfortunately, there are no sufficient studies. However, I have a lot of concerns regarding this study as it requires further deep investigations, I still recommend its publication as it could shed the light in this direction.

1- What is the 1ry antibody used in the western blot

- The first antibody used was anti-ExoS, the information was included in Materials and methods and in the figure legends.

2- I think it would be nice if you can quantify the expresed proteins (use flourescent 2ry antibody and quantify the flourecence, for instance) to obtain statistical significance

- Thank you for your suggestion. We carried out a densitometry analysis to obtain statistical significance using at least three Western blot assays and it was included in the Western blot figures. Also, the methodology was included (lines 264-268).

3- Line 52, please re-write "Mutant strains defective in this system are severely attenuated in their virulence" with clarification

- Thanks for your suggestion. The sentence was modified for clarification (lines 51-52)

4- Please clarify the methodology section to be more clear to non-experts, as clarifying the importance of Groel protein as reference one

- The methodology section was reviewed and modified to be clear.

5- The discussion is well understood, however I recommend writing more detailed conclusion.

- We have written a more detailed conclusion according to your suggestion (lines 562-565)

6- I may recommend the authors to draw a represenative graph illustrating their findings

- Thank you for your recommendation. A figure summarizing our findings has been included (Fig 9).

7- Furthermore, I may advise the authors to extend their findings showing the impact on the bacterial virulence in vitro by quantification of pyocyanin as an example to QS or immunstining to the exoS proteins in macrophages or HeLa cells, as an example

-	As you suggested, we quantified pyocyanin production in T3SS-induction conditions. This was included in the revised manuscript (lines 258-262 and 458-476).

Reviewer #2: This paper examines the role of the global regulator RhlR in the regulation of Pseudomonas aeruginosa type 3 secretion system (T3SS). The transcriptional regulator RhlR was initially characterised at the end of the last century as the receptor for the quorum-sensing signal molecule: N-butanoyl-L-homoserine lactone (C4). However, it is now known that this protein has multiple mechanisms of action, both C4-dependent and C4-independent. The authors re-examine what the role of QS might be in the regulation of T3SS in light of today's knowledge.

Briefly, by means of transcriptional fusions and western-blot experiments, evidence is brought to support the hypothesis that RhlR positively regulates the expression of ExoS (the main effector of T3SS) in a C4-independent manner. Preliminary evidence is also brought to support the hypothesis that RhlR can regulate exoS transcription by positively controlling the promoter of the exsCEBA operon, which in turn encodes for the complex regulatory system that controls T3SS gene expression.

Overall, the authors show that: i) RhlR positively regulates the expression of exoS regardless of the presence of the C4 inducer; ii) this mechanism involves activation of the exsCEBA operon promoter; iii) constitutive transcription in trans of exsA, the main activator of T3SS, restores the expression of exoS in a rhlR mutant.

This is interesting work because it disproves the common belief that QS negatively regulates T3SS expression and draws attention to the multiple mechanisms by which RhlR can control P. aeruginosa virulence. Unfortunately, the work remains somewhat superficial and does not go deeper into the mechanism underlying the RhlR-dependent regulation of exoS and exCEBA genes.

Overall, the work is a bit preliminary and should be enriched with further experiments. Below there are some major issues that the authors need to address in order to get the work published:

1) The regulation of T3SS is very complex, the authors should explain it a little better in the introduction, possibly by including a figure in supplementary showing the exsCEBA operon and the exoS and spcS genes, also indicating the promoters cloned into transcriptional fusions with the lux genes. Concerning T3SS, it would be useful to cite Horna & Ruiz's review (doi:10.1016/j.micres.2021.126719).

- Thank you for your suggestion. In this revised version we have included two additional supplementary figures related to the regulation of the T3SS and the transcriptional fusions constructed in this work (Fig S1 and S2). Also, the cite Horna and Ruiz was included.

2) In Figures 1, 2 and 3, it is not clear whether the experiments were conducted under inducing or not-inducing conditions. In any case, the authors should conduct the experiments shown in Figures 1, 2 and 3 under both conditions.

- All experiments were conducted under inducing conditions since in LB the T3SS is not active. We have included this information in the revised version and also, we have included a supplementary figure where we showed that ExoS is not detected in non-induction conditions (lines 281-282, 498-500) (Fig S6).

3) The names given to transcriptional fusions (pS, pP, pC, pA) are too contracted and do not help understanding. Authors should name the promoters as per convention (es. promoter of exoS gene = PexoS. Fusion between PexoS and luxABCDE = PexoS::luxABCDE, the latter could be abbreviated as PexoS::lux).

- Names of plasmids were changed in the text according to your suggestion.

4) The PexsCEBA promoter is activated by RhlR in a C4- and PqsE-independent manner. Thus, this regulation appears to be indirect. The authors speculate that the regulation might depend on the spermidine binding protein PA2592 (lines 457-463). The authors should construct this mutant and test this hypothesis.

- In this work, our main objective was to define the role of the quorum sensing systems on the T3SS expression. Therefore, we did not determine the target of RhlR to control the T3SS expression. However, according to your suggestion, in this revised version, we explored whether PA2595 has a role in the T3SS control. Our approach was to evaluate whether the expression of *PA2592* under a constitutive promoter was able to restore ExoS secretion in the *rhlR* mutant strain. These results were included as a new section (lines 432-456). Also, the discussion was modified according to these results (lines 551-560).

5) The PexsCEBA promoter is regulated by other regulators such as PrsA/RpoS, ArtR, RtsM (Horna & Ruiz of 2021 and references therein). Is there a relationship between these regulators and RhlR? The authors should answer this question referring to literature data or by conducting experiments.

- Thank you for your comment. There is not a relationship previously reported between RhlR and PsrA, ArtR, or RetS (RtsM). Also, we searched for a *las*-box in these and other regulators of the T3SS reported by Horna & Ruiz but we could not find any consensus sequence for RhlR. We discussed these findings in the discussion section (lines 547-551).

6. PLOS authors have the option to publish the peer review history of their article (what does this mean?). If published, this will include your full peer review and any attached files.

If you choose “no”, your identity will remain anonymous but your review may still be made public.

Do you want your identity to be public for this peer review? For information about this choice, including consent withdrawal, please see our Privacy Policy.

Reviewer #1: Yes: Wael A. H. Hegazy

Reviewer #2: No
