# Supplementary material for: The quorum sensing regulator RhlR positively controls the expression of the type III secretion system in Pseudomonas aeruginosa PAO1
Source: PLoS One. 2024 Aug 15;19(8):e0307174. doi: 10.1371/journal.pone.0307174 (PMC11326643; doi:10.1371/journal.pone.0307174)
Supplement: Attachment — Submitted filename: Response to Reviewers.docx [file pone.0307174.s014.docx]

June 17, 2024

Rajesh P. Shastry, Ph.D

Academic Editor

PLOS ONE

In this document, we respond to the comments and suggestions made by the reviewer for the manuscript PONE-D-24-00964R1 entitled ‘The quorum sensing regulator RhlR positively controls the expression of the type III secretion system in *Pseudomonas aeruginosa* PAO1’ by Luis Fernando Montelongo-Martínez, Miguel Díaz-Guerrero, Verónica Roxana Flores-Vega, Martín Paolo Soto-Aceves, Roberto Rosales-Reyes, Sara Elizabeth Quiroz-Morales, Bertha González-Pedrajo, Gloria Soberón-Chávez, Miguel Cocotl-Yañez.

Best regards

Miguel Cocotl-Yañez

Corresponding author.

Reviewers' comments:

Reviewer's Responses to Questions

**Comments to the Author**

1. If the authors have adequately addressed your comments raised in a previous round of review and you feel that this manuscript is now acceptable for publication, you may indicate that here to bypass the “Comments to the Author” section, enter your conflict of interest statement in the “Confidential to Editor” section, and submit your "Accept" recommendation.

Reviewer #1: All comments have been addressed

Reviewer #3: (No Response)

2. Is the manuscript technically sound, and do the data support the conclusions?

The manuscript must describe a technically sound piece of scientific research with data that supports the conclusions. Experiments must have been conducted rigorously, with appropriate controls, replication, and sample sizes. The conclusions must be drawn appropriately based on the data presented.

Reviewer #1: Yes

Reviewer #3: Yes

3. Has the statistical analysis been performed appropriately and rigorously?

Reviewer #1: Yes

Reviewer #3: Yes

4. Have the authors made all data underlying the findings in their manuscript fully available?

The [PLOS Data policy](http://www.plosone.org/static/policies.action#sharing) requires authors to make all data underlying the findings described in their manuscript fully available without restriction, with rare exception (please refer to the Data Availability Statement in the manuscript PDF file). The data should be provided as part of the manuscript or its supporting information, or deposited to a public repository. For example, in addition to summary statistics, the data points behind means, medians and variance measures should be available. If there are restrictions on publicly sharing data—e.g. participant privacy or use of data from a third party—those must be specified.

Reviewer #1: Yes

Reviewer #3: Yes

5. Is the manuscript presented in an intelligible fashion and written in standard English?

PLOS ONE does not copyedit accepted manuscripts, so the language in submitted articles must be clear, correct, and unambiguous. Any typographical or grammatical errors should be corrected at revision, so please note any specific errors here.

Reviewer #1: Yes

Reviewer #3: Yes

6. Review Comments to the Author

Please use the space provided to explain your answers to the questions above. You may also include additional comments for the author, including concerns about dual publication, research ethics, or publication ethics. (Please upload your review as an attachment if it exceeds 20,000 characters)

Reviewer #1: (No Response)

Reviewer #3: In this MS Cocotl-Yanez et al. delve into the role of QS in regulating T3SS in Pseudomonas aeruginosa. Since QS has branching global effects on virulence in this pathogen, this is a very relevant topic and the results provided by this group shed some light on T3SS regulation and provide highly needed clarifications to some experimental discrepancies found regarding this topic. The authors make a sound analysis on the regulation of the T3SS and provide solid data that support most of their conclusions. Although I consider it a high quality work and I would support this MS for its acceptance, there are a few concerns that I think need to be addressed regarding some of their conclusions:

Major concerns:
The authors conclude that, as overexpressing exsA restores ExoS production in a rhlR mutant, RhlR controls the T3SS expression via ExsA. This is not necessarily the case, they can both act independently, and overexpressing one of the two independent activators may still correct the lack of the other. Their data proves that RhlR does not act downstream of ExsA, but there is still the question if RhlR acts upstream of ExsA as they claim or if they act in different signaling pathways. This would be clarified if they introduced their rhlR overexpression plasmid in a exsA mutant background. If overproducing RhlR can also restore ExoS production they might be independent activators. On the other hand, if ExoS production is not restored, it may indicate that both regulators act hierarchically as they describe.

R: Thank you for your suggestion. We overexpressed *rhlR* in a strain defective in the T3SS and transcription of *exsCEBA* and *exoS* were measured, the results are described in lines 378-387.

I have trouble relating ExoS quantifications in figure 5b with their associated western blot data. What I see in the graphs is not an increase in ExoS protein levels in the rhlI mutant background as claimed in the main text (L399) and glimpsed in the western blot (in any case average levels in the graphs seem to be higher in the pqsE mutant although not significant). Also the statistical analysis does not seem to indicate that any of the mutants besides rhlR is significantly different from the wild type to make any of these statements. Please clarify these discrepancies.

R: Thank you for your observation. We have corrected it (lines 416-417) and also it was discussed (lines 556-561).

Minor concerns:
Also regarding ExsA and RhlR relationship, it would be interesting to see if the internal PexsA promoter is also affected by the rhlR mutation.

R: Thanks for your comment. We found that *exsA* internal promoter is barely expressed and is not activated by induction conditions, thus we focused on determining the role of the QS systems on the T3SS genes that were activated in induction conditions.

L118: I fail to understand why are there discrepancies on T3SS regulation by the Las system. It is clear why Rhl role in T3SS needs further clarification, but all evidence discussed up to this point about Las system seems to agree that it is not involved. I would suggest changing this to “there are some discrepancies in the virulence and regulation of the T3SS by QS systems” so it is more accurate.

R: It was changed (Line 118)

L302: The first section of the results jumps from figure 1 to figure 3. If the authors consider that discussing this mutant at this point of the MS is important I would suggest to rename it as figure 2. Otherwise they can try to introduce this result at a later point.

R: Figure 3 was renamed as Figure 2.

L395: As they also mentioned in the introduction, RhlR is known to be able to act independently of C4. I would rephrase this to “can be dependent” or other formulation that better reflects that this is not an absolute dependence.

R: It was changed (Line 414)

L401-404: It is a bit hard to understand why this result is discussed here, and it also introduces a bit of difficulties in following the reasoning of the experimental procedure. I would move this phrase to a different point, maybe after discussing the results on Fig 6, so it is easier to follow and shows in a clear way why there are differences between exsC promoter activities in Fig 6 and S5.

R: The section was modified according to your suggestion.

L438, 442, 551, and 553: The authors probably mean a rhl box or a las-rhl box?

R: Thank you. It was changed to las-rhl box.

L461: The authors should clarify how their results show dependence on C4 and PqsE. By looking at figure 8 one can see PqsE dependence, but this is not discussed in the text, and C4 dependence is equally not properly described in this section despite being shown in the picture. Please reformulate this whole section so it is clear to the reader why your data supports your claims (which does).

R: Thank you for your comment. The section was modified and also the results were discussed (Lines 586-591).

Minor errata and things the authors may want to check before submitting their final version:
L73: control. R: Corrected
L127: show. R: Corrected
L170: remove the primer sequence since it is already in the supplementary table and no other primer sequences are shown. R: Corrected
L265: A citation may be missing here for “as reported previously”. R: Corrected

7. PLOS authors have the option to publish the peer review history of their article ([what does this mean?](https://journals.plos.org/plosone/s/editorial-and-peer-review-process#loc-peer-review-history)). If published, this will include your full peer review and any attached files.


If you choose “no”, your identity will remain anonymous but your review may still be made public.


**Do you want your identity to be public for this peer review?** For information about this choice, including consent withdrawal, please see our [Privacy Policy](https://www.plos.org/privacy-policy).

Reviewer #1: **Yes:** Wael A. H. Hegazy

Reviewer #3: **Yes:** Francisco Javier Marcos-Torres
